# Aggression and Affiliation during Social Conflict in Pigs

**DOI:** 10.1371/journal.pone.0113502

**Published:** 2014-11-26

**Authors:** Irene Camerlink, Simon P. Turner, Winanda W. Ursinus, Inonge Reimert, J. Elizabeth Bolhuis

**Affiliations:** 1 Adaptation Physiology Group, Department of Animal Sciences, Wageningen University, Wageningen, The Netherlands; 2 Animal Breeding and Genomics Centre, Department of Animal Sciences, Wageningen University, Wageningen, The Netherlands; 3 SRUC, Edinburgh, United Kingdom; 4 Animal Behaviour & Welfare, Wageningen UR Livestock Research, Wageningen, The Netherlands; Brock University, Canada

## Abstract

Social conflict is mostly studied in relation to aggression. A more integral approach, including aggressive and affiliative behaviour as well as physiology, may however give a better understanding of the animals' experience during social conflict. The experience of social conflict may also be reflected in the spatial distribution between conspecifics. The objective was to assess the relationship between behaviour, physiology, and spatial integration in pigs (*Sus scrofa*) during social conflict. Hereto, 64 groups of pigs (9 wk of age) were studied in a 24 h regrouping test whereby pairs of familiar pigs were grouped with 2 unfamiliar pairs, in either barren or straw-enriched housing. Data on aggressive and affiliative behaviour, skin lesions, body weight, and haptoglobin could be summarized into three principal component analysis factors. These three factors were analysed in relation to spatial integration, i.e. inter-individual distances and lying in body contact. Pigs stayed up to 24 h after encounter in closer proximity to the familiar pig than to unfamiliar pigs. Pigs with a high factor 1 score were more inactive, gave little social nosing, had many skin lesions and a high body weight. They tended to space further away from the familiar pig (*b* = 1.9 cm; *P* = 0.08) and unfamiliar ones (*b* = 0.7 cm; *P* = 0.05). Pigs that were involved in much aggression (factor 2), and that had a strong increase in haptoglobin (factor 3), tended to be relatively most far away from unfamiliar pigs (*b* = 0.03 times further; *P* = 0.08). Results on lying in body contact were coherent with results on distances. Pigs in enriched housing spaced further apart than pigs in barren housing (*P*<0.001). The combined analysis of measures revealed animals that may either promote or slow down group cohesion, which may not have become clear from single parameters. This emphasizes the importance of an integral approach to social conflict.

## Introduction

When unfamiliar animals first meet this may result in social conflict. Social conflict may involve excessive aggression, and efforts have been made to reduce aggression in captive animals [1] [2]. Aggression, however, only resembles part of the behavioural repertoire that animals may express to solve social conflicts. Alongside aggression there may be subtle affiliative behaviours, such as social grooming and body contact, that may promote reconciliation and group cohesion [3], [4]. These behaviours are well studied in primates [5], but largely ignored in most other species [6], [7], [8]. Assessing animals, for example for breeding purposes or welfare classification, should not only require assessment of aggression, but might also require taking into account cognitive processes and social skills that may promote social cohesion (pigs [2], crested macaque [9]).

In gregarious animals, social cohesion may be measured by the distance between group members (i.e. their spatial integration) and their affiliative interactions such as allogrooming ([10], marmots [11], pigs [12]). These measures may reflect social acceptance, and have been suggested to reflect the animals' experience of a stressful situation [5], [12]. Animals may show clear preferences for whom they allow in close proximity and will move away or show aggression when disliked animals approach too closely [13], [14]. This is especially relevant in livestock farming, where animals may be introduced to unfamiliar conspecifics several times during the production cycle and are commonly kept under minimal space requirements, allowing little room to move away from each other (pigs [15], poultry [16]).

In commercially kept pigs, regrouping aggression is considered to be a major welfare issue [17]. Regrouping, whereby unfamiliar animals encounter each other, is a common management strategy in commercial pig farming to obtain groups of equal body weight or gender. Regrouping results in aggressive interactions, which amongst others causes (skin) injuries [18], [19], and alters stress physiology, e.g. [20]. Aggression in pigs has been studied for decades [1], [21], but up to now remains a persistent problem, which may emphasize the need for a different approach. Insight into pigs' experience of social conflict might contribute to a solution, but this has hardly been studied [12], [22].

To solve complex behavioural issues, and to achieve sustainable welfare improvement, an integral approach might be required [23]. We aimed to take such an approach in pigs by assessing behavioural and physiological characteristics that may be related to the spatial integration between pigs during social conflict. We hypothesized that the spatial integration of regrouped pigs would depend on aggressive interactions as well as on positive social contact. The objective was to investigate if specific characteristics in behaviour and physiology would contribute most to small inter-individual distances, indicative of social cohesion.

## Materials and Methods

This study was part of a larger trial in which animals were selected for diverging indirect genetic effects for growth (IGEg) and either housed in barren or enriched pens (described in *Animals and housing*). Because these factors may influence the social interactions between animals, they were taken into account in the current study.

### Ethics

This study was carried out in strict accordance with the recommendations in the European Guidelines for accommodation and care of animals. The protocol was approved by the Institutional Animal Care and Use Committee of Wageningen University (Protocol Number: 2010055).

### Animals and housing

A total of 384 pigs of 9 weeks of age, housed in 64 pens, were studied over four batches. These animals were offspring from 64 TOPIGS-20 sows (sow line of Great Yorkshire × Dutch Landrace) and 24 Tempo boars (commercial synthetic boar line with Great Yorkshire genetic background), which were selected on either ‘high’ or ‘low’ indirect genetic effects for growth (IGEg). IGEg is a breeding value that accounts for the genetic effect that a pig may have on the growth rate of its group members [24], [25], [26]. Details of the selection on IGEg are previously described [27]. Piglets were housed in conventional farrowing crates until weaning (at 26 days of age).

From weaning until slaughter, pigs were housed in groups of six (three females and three castrated male pigs) of the same IGEg classification. Each group contained at least one pig from both sexes that had an active response in the backtest, with a minimum of two pigs from the same backtest classification [27]. The backtest is a behavioural test whereby the response of piglets may be indicative of a piglet's coping style or behavioural strategy [28], and may relate to its aggressive behaviour as well as to other traits [29].

In the 2×2 experimental arrangement that was applied from weaning, half of each IGEg group (high vs. low) was housed in barren pens and the other half was housed in enriched pens. The barren conventional pens had a 60% solid concrete and 40% slatted floor. Enriched pens had a solid floor with a deep litter bedding of straw and wood shavings. Space allowance in both housing conditions was between 1.0–1.2 m^2^ per pig, depending on the barn. Pen dimensions were 1.90 m×3.20 m in two batches, whereas in the other two batches pens measured 2.25×3.25 m. Each pen contained a single space feeder, a nipple drinker, and a metal chain with a ball. Pigs were fed *ad libitum* with dry pelleted feed. The thermostat was set at 20°C, and minimum and maximum ambient temperature were recorded daily. Lights were on between 07:00 and 19:00 h.

### Regrouping test and skin lesion score

At nine weeks of age, pigs were regrouped for 24 h (∼12:00 h to ∼12:00 h the following day) within their IGEg group and housing condition. From each pen, one male and one female pig were relocated into an unfamiliar pen where they were joined with two pairs of pigs originating from two different pens. None of the pigs in the new pen were full-sibs, and pen composition was balanced for backtest response. Pigs received a number sprayed on their back for recognition, and a coloured dot on their neck (stock marker spray) to identify the pig pair. Video cameras were mounted above the pens to enable video recordings. After 24 h, pigs were returned to their initial pen, and the number of fresh skin lesions was counted for the anterior, middle, and rear regions for both sides of the body immediately upon return [18]. For each pig, body weight was recorded at 9 weeks of age.

### Spatial distribution

Data on spatial distribution of pigs were obtained from the video footage that was available from the regrouping test. For each pen there were video recordings from 11:00–19:00 h on the day of regrouping, and from 07:00–12:00 h the following day. Every hour, from the moment that all six pigs had entered the new pen, a screenshot was made from the video footage. The screenshot was made when at least 4 of the 6 pigs were lying. In case more than 2 pigs were standing, the video was forwarded until the moment that at least 4 pigs were lying. In this way, 13 to 14 images were obtained per pen. For each pen, a grid with corresponding x and y coordinates was made at an appropriate scale to be overlaid on the video playback. Hereto, the screenshot was focussed on one pen and rulers were placed along the sides of the pen. Rulers were adapted for scale to fit the true pen dimensions and depth, whereby each cm on the ruler represented 20 cm in reality. From the rulers, the x and y coordinate at the height of the neck of each pig was noted. When the neck of a pig could not be located due to objects in front of the lens (e.g. feeder), the middle of the pig was taken as a reference point. When the distances were calculated, a distinction was made between the familiar pig of the pair and the 4 unfamiliar pigs. It was also noted whether a pig was lying with at least 50% of its body in direct contact with a familiar or unfamiliar pig, whereby the middle of the pig was used as a reference point.

### Blood collection and haptoglobin determination

Haptoglobin is an acute phase protein that may reflect amongst others immune activation and has been suggested as potential biomarker for stress [30], [31], [32], [33], also in pigs [34], [35], [36]. Pigs were blood sampled in the week before the regrouping test (wk 8) and at the third day after the test (wk 9), as haptoglobin may peak several days after the initial stressor, e.g. [37], [38], [39]. Blood was drawn by puncture of the jugular vein. The order of sampling was randomized over IGEg group and housing condition. Blood was collected in a serum tube and stored at room temperature. The samples were incubated for one hour at 37°C, and thereafter centrifuged at 20°C at a speed of 5251 g for 12 min. The serum obtained was stored at −80°C. The haptoglobin concentration was determined from the serum using a commercial kit based on the hemoglobin-binding capacities of haptoglobin (PhaseTM Haptoglobin, Tridelta Development Limited, Maynooth, Ireland), which has been validated for pigs (GD Animal Health Service, Deventer, the Netherlands). Hemoglobin (100 µl) was added to sera (7.5 µl) and gently mixed. Thereafter, chromogen (140 µl) was added and the solution was incubated for 5 min at room temperature and the absorbance read immediately at 600 nm in a microplate reader. The concentration of haptoglobin (mg/ml) was calculated with a standard linear curve for known concentrations of haptoglobin. The difference between the basal level and the level following regrouping (wk 8 subtracted from the levels at wk 9) was used for analyses, and is here referred to as Δhaptoglobin.

### Live behavioural observations

Behaviour of the pigs was observed live during the regrouping test by 2-min instantaneous scan sampling for six hours. The Observer 5.0 software package (Noldus Information Technology B.V., Wageningen, The Netherlands) installed on a hand-held computer was used for behaviour recordings. Observations were divided into 1 h blocks (with 15 min breaks between each block) from 14:00–17:30 h on the day of regrouping and from 08:00–11:30 h the following day. This procedure resulted in 180 observations per pig. Behaviours that were analysed were inactivity (lying with the eyes closed or eyes open), aggression (reciprocal fighting, head knocks, and unilateral biting), and social nosing (nosing the body of a pen mate without performing oral manipulation and nose-nose contact with a pen mate). For aggressive behaviour and social nosing both the giver and receiver of the behaviour were recorded as either a familiar pig or an unfamiliar one.

### Data preparation

The distance between two spatial coordinates was calculated using a^2^+b^2^ = c^2^, in which a = x_pig1_-x_pig2_, where x_pig1_ is the x-coordinate of one pig and x_pig2_ the x-coordinate of another pig. The same procedure was used for b, which is the difference between the y-coordinates of two pigs. The square root of the resulting c^2^ was the distance between two pigs.

The distances to the four unfamiliar pigs were averaged, resulting in one value for the distance to the familiar pig and one value for the average distance to the unfamiliar pigs. Due to the lying positions of the pigs, whereby the back of the pig was not always visible from the cameras' perspective, the individual markings on the back of the pigs could not always be identified. The specific colour marking for pig pairs (marking in the neck) could, however, always be identified and therefore the average distance to unfamiliar pigs was only available per pig pair. Pig pairs where one of the pigs was removed from the experiment (due to health reasons prior to the regrouping test) were excluded from analysis (*n* = 5). A relative distance between familiar and unfamiliar pigs was calculated by dividing the average distance to the unfamiliar pigs by the distance to the familiar pig. The original data from all observations on distances (S1) and by pig (S2) are available in the supplementary files.

Residuals of the variables were obtained from a general linear model (Proc GLM) with housing condition, IGEg group, and batch as fixed effects. For Δhaptoglobin one outlier was removed (3.2 mg/ml), which was 4.4 SD higher than the mean (0.14 mg/ml) and 1.3 SD higher than the second highest value. The skin lesion scores were square root transformed, and the behavioural observations were transformed by arcsine square root. After transformation, all residuals followed a normal distribution.

### Principal component analysis (PCA)

**Figure 1:**
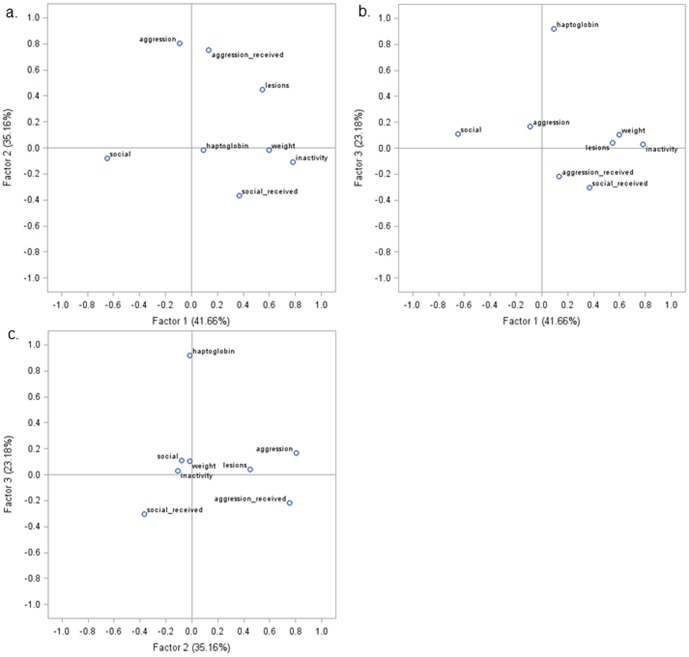
Factor maps of the PCA. Distributions in relation to the principal component factor 1 and 2 (A), factor 1 and 3 (B), and factor 2 and 3 (C) extracted after principal component analyses (PCA). Loadings of each variable on the principal component serve as coordinates on the X-axis and Y-axis, respectively.

The behavioural and physiological data were correlated with Pearson correlations on the residuals of the GLM. Four out of the 10 variables showed at least one significant correlation above 0.30. Data were therefore analysed in a principal component analysis (PCA) with orthogonal rotation. The skin lesions on the anterior, middle, and rear of the body were strongly correlated (r_p_ 0.45–0.73; all *P*<0.001). To prevent creating a PCA factor that would only resemble skin lesions due to the high correlations, we included the total number of skin lesions in the PCA rather than the numbers by body region. The PCA resulted in three factors with an Eigenvalue above 1. The loadings on the factors are given in Table 1 (*Results* section) with the corresponding factor maps (Figure 1).

**Table 1:** Loadings on the factors extracted by the principal component analysis, after orthogonal rotation, of variables recorded on individual pigs.

Measure	Factor 1	Factor 2	Factor 3
Skin lesions	**0.55**	*0.45*	0.04
Body weight	**0.60**	−0.02	0.10
ΔHaptoglobin	0.09	−0.02	**0.92**
Aggression given	−0.09	**0.80**	0.16
Aggression received	0.13	**0.75**	−0.22
Social nosing given	**−0.65**	−0.08	0.11
Social nosing received	*0.37*	*−0.36*	−0.30
Inactivity	**0.78**	−0.11	0.03
Eigenvalue	1.85	1.56	1.03
Variance explained (%)	23.5	19.4	12.8

Values between 0.30–0.50 are in italics, values above 0.50 are in bold.

### Data analysis

Data preparation, the PCA, and data analysis were carried out in SAS 9.2. The effect of familiarity on the spatial integration between pigs was analysed in a mixed model (Proc MIXED) with repeated measures per pair (1).

(1) Spatial integration_pair_  =  familiarity + IGEg group + housing condition + batch + pig pair (pen + IGEg group + housing condition + batch) + pen + *e*, where ‘spatial integration’ denotes distance and body contact on pair level. ‘Familiarity’ was a class effect (unfamiliar/familiar) resulting in two repeated values per pair. Pig pair was included as a random effect to account for the repeated measures per pair, and was nested within pen, IGEg group, housing condition, and batch. Pen was included as second random effect.

The difference in distance between familiar and unfamiliar pigs over time was analysed in a mixed model (2) with 14 repeated observations over time per pair.

(2) Difference in distance_pair_  =  observation + IGEg group + housing condition + batch + pig pair (pen + IGEg group + housing condition + batch) + pen + *e*, where ‘difference in distance’ was the distance to unfamiliar pigs minus the distance to the familiar pig. ‘Observation’ (∼14 observations over 24 h) was included as fixed class effect, while pig pair was included as random effect nested within pen, IGEg group, housing condition, and batch, along with the random pen effect. Two- and three way interactions were tested, but omitted from the model due to non-significance.

The effect of behaviour and physiology on the spatial integration between pigs was analysed using a mixed model (3), with the response variables separate for familiar and unfamiliar pigs.

(3) Spatial integration_pair_  =  factor 1_pig_ + factor 2_pig_ + factor 3_pig_ + IGEg group + housing condition + batch + pig pair (pen + IGEg group + housing condition + batch) + pen + *e*, where ‘spatial integration’ denotes the measures on pair level for the distance to the familiar pig, the average distance of a pig pair to unfamiliar pigs, the relative distance between the two, and the proportion of observations that pigs spent in body contact with a familiar or an unfamiliar pig. The three PCA factors were on individual pig level and were included as explanatory variables. Interactions between these variables were explored and significant interactions were retained. The effect of IGEg group, housing condition, and batch on the spatial integration was explored, along with the relevant interactions between these variables. The pig pair and pen were included as random effects, whereby pig pair was nested within pen, IGEg group, housing condition, and batch. The residuals of model 1 and 3 were normally distributed for all spatial integration variables.

The difference in haptoglobin concentration was tested in a mixed model with the sample moment (pre-test/post-test) as repeated observation. The haptoglobin concentration was the response variable whereas ‘sample moment’, IGEg group, housing condition, and batch were explanatory variables, with the pig pair and pen as random statements as described above.

Values presented are (untransformed) LSmeans ± SEM.

## Results

### Familiarity

**Figure 2:**
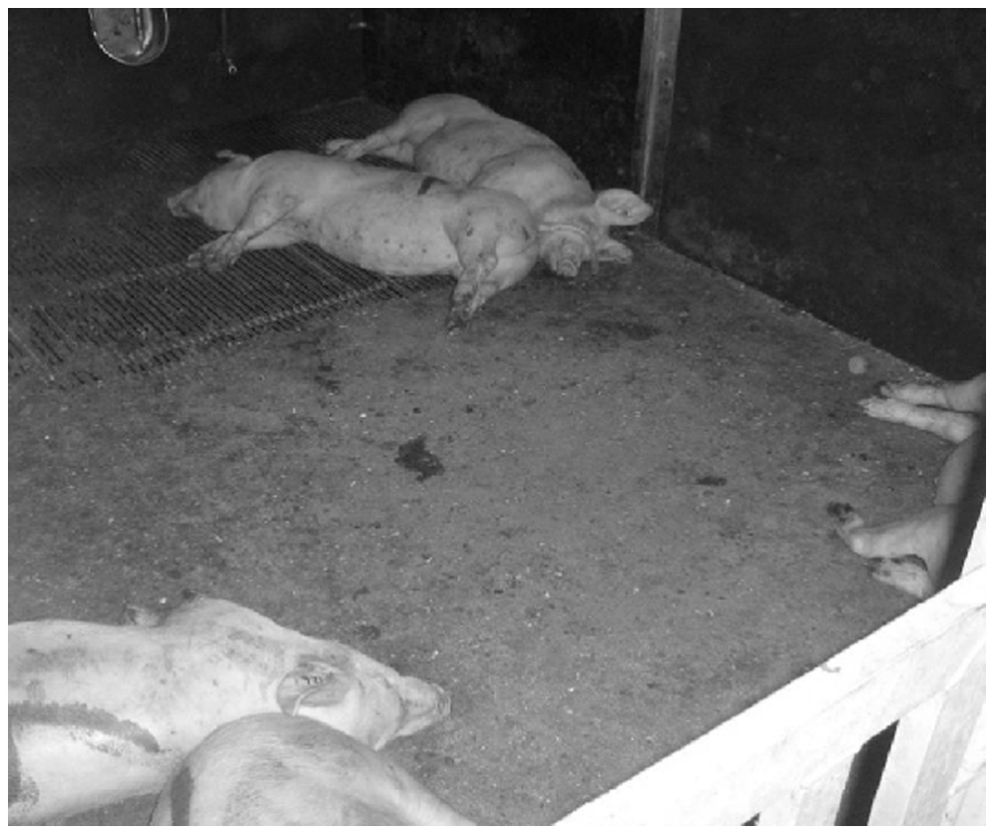
Pigs resting closely together with their familiar pig while resting far apart from unfamiliar pigs.

**Figure 3:**
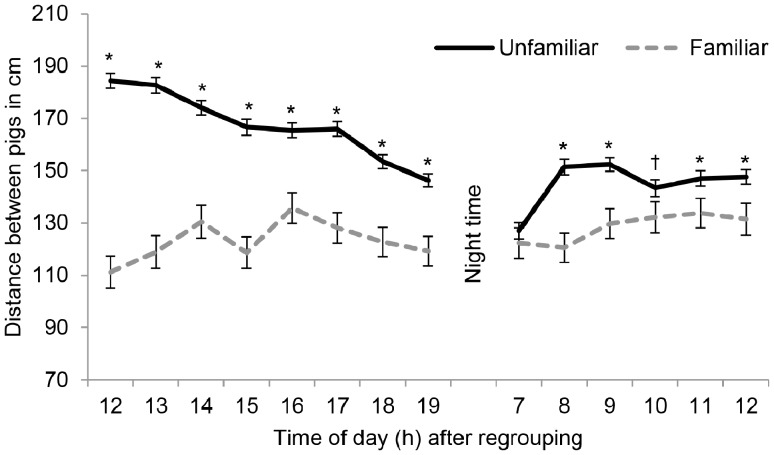
Familiarity affects distance till at least 24 h after encounter. Distance between familiar pigs and their average distance to the four unfamiliar pigs over the course of 24 hour after regrouping, *n* = 192 pig pairs.

Pigs stayed in close proximity to the pig they were familiar to, whereas they spaced away from unfamiliar pigs (Figure 2). On average, familiar pigs lay 125±2 cm apart, whereas their average distance to unfamiliar pigs was 158±2 cm (*F*_1,187_ = 205; *P*<0.001). Pigs stayed in closer proximity to the familiar pig than to the unfamiliar pigs until night time (Figure 3; all *P*<0.001). On the following morning, the distance between unfamiliar pigs was smaller, but remained significantly larger than the distance between familiar pigs (all *P*<0.05), except at 07:00 h (*P* = 0.39), which was the first hour after the dark period, and at 10:00 h (*P* = 0.07).

The closer proximity between familiar pigs was also reflected in the amount of body contact. Familiar pigs lay with at least half of their body in direct contact with each other on an average of 14±0.7% of the observations (range 0–71%), but in contact with one or more of the unfamiliar pigs on 21±0.7% of observations (range 0–54%). If pigs did not differentiate between familiar and unfamiliar pigs when resting, the chance that a pig would lie next to the familiar pig would be four times smaller (^1^/_5_) than to one of the unfamiliar pigs (^4^/_5_). When corrected for chance, the frequency of lying in body contact with an unfamiliar pig would equate 5±0.7%. Therefore, pigs lay in contact with the familiar pig more often than with unfamiliar pigs as would be expected by chance (*P*<0.001).

### Principal component analysis

The principal component analysis revealed three factors, which together explained 55.6% of the variation (Table 1). A high score on factor 1 related to inactivity, a low amount of giving social nosing towards others, a high number of skin lesions, and a high body weight. This combination of variables might describe sociality. A high score on factor 2 was mainly explained by a high amount of aggressive behaviour given and received, as well as a high number of skin lesions. Factor 2 described involvement in aggression (not ‘aggressive’ in itself, because it also includes pigs that received much aggression and are therefore not necessarily aggressive themselves). Factor 3 explained the increase in the level of haptoglobin, i.e. Δhaptoglobin. The increase in haptoglobin was unrelated to the amount of skin lesions (r_p_ 0.07; *P* = 0.21), which was also reflected in the loadings in factor 3 (Table 1). The receipt of social nosing was the only variable that did not clearly associate with one of the factors (Table 1). Separate analysis of this variable in the mixed models indeed did not reveal a relationship with measures on the distances or body contact.

### Inter-individual distances

Pigs with a high factor 1 score tended to space further away from the familiar pig (*b* = 1.9±1.1 cm; *P* = 0.08), as well as from unfamiliar pigs (*b* = 0.7±0.4 cm; *P* = 0.05). Opposite, animals that were active, involved little in aggression, and gave much social nosing, i.e. the animals with a low factor 1 score, were closely situated to other pigs. Pigs spent 1.8±1.3% of the behavioural scans giving social nosing, of which on average 20% was directed towards the familiar conspecific.

Pigs with a high factor 2 score gave and received high amounts of aggressive behaviour. Aggressive behaviour, which occurred on average on 1.1±1.4% of the behavioural scans observed after the first peak of aggression, was directed towards unfamiliar pigs on 90% of occasions. Involvement in aggression (factor 2) did not significantly affect distances toward the familiar pig (*b* = −0.9±1.1 cm; *P* = 0.46) or toward unfamiliar pigs (*b* = 0.1±0.4 cm; *P* = 0.87).

Increased haptoglobin (factor 3) did not affect the distance toward the familiar pig (*b* = −1.1±1.1 cm; *P* = 0.31) or unfamiliar pigs (*b* = −0.4±0.4 cm; *P* = 0.24). The haptoglobin concentration significantly increased after regrouping (*P*<0.001). Before the test the haptoglobin concentration was on average 0.55±0.03 mg/ml (0.04–2.55), and at 3 d after the test it was on average 0.70±0.03 mg/ml (range 0.04–3.5), resulting in a difference (Δ) of 0.14±0.03 mg/ml (range −2.0–2.3). These values are within a normal range for healthy animals.

### Relative distance

Pigs were on average 1.3±0.02 times further away (range 0.8–3.3) from unfamiliar pigs than from the pig they were familiar to, which is here referred to as the relative distance. Although pigs with a high factor 1 score (sociality) spaced further away from the unfamiliar pigs as well as from the familiar pig, this was only in absolute distance, and not relative to each other (*P* = 0.47). Since both absolute distances were increased, the relative distance to unfamiliar pigs as compared to the distance to the familiar pig remained the same. The relative distance tended to be greatest, i.e. pigs were relatively most far away from unfamiliar pigs, in pigs with a high factor 2 score (much involvement in aggression) and a high factor 3 score (high increase in haptoglobin) (*b* = 0.03±0.01 times further; *P* = 0.09). The relative distance to unfamiliar pigs was not affected by the separate contribution of factor 2 (*b* = 0.02±0.02 times further; *P* = 0.29) or factor 3 (*b* = 0.01±0.01 times further; *P* = 0.41).

### Lying in body contact

Pigs with a high factor 2 (much involvement in aggression) and a high factor 3 score (high increase in haptoglobin) tended to lay most in body contact with the familiar pig (*b* = 0.8±0.4% of observations; *P* = 0.09), but less with unfamiliar pigs (*b* = −0.4±0.3% of observations; *P* = 0.10). Lying in body contact with the familiar pig or with unfamiliar pigs was unaffected by factor 1 (*P* = 0.94; *P* = 0.48, respectively), or the separate contribution of factor 2 (fam: *P* = 0.99; unfam: *P* = 0.86) or factor 3 (fam: *P* = 0.69; unfam: *P* = 0.82).

### Enriched housing conditions

**Figure 4:**
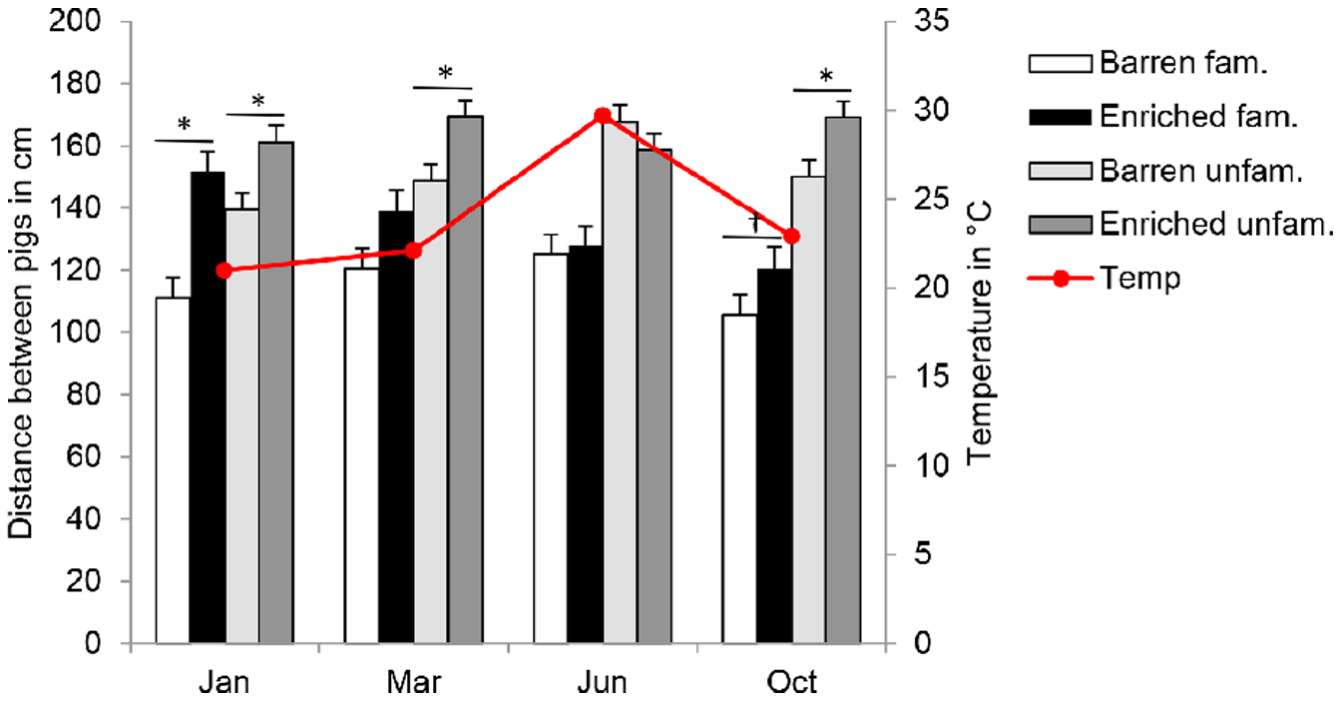
Larger distances in enriched housing conditions. Distances between familiar and unfamiliar pigs in barren and enriched pens, presented by the month in which regrouping took place (batch). The secondary axes shows the maximum temperature in the barn at the day of regrouping.

Pigs in pens that were enriched with a deep litter layer of straw and wood shavings kept a larger distance to familiar and unfamiliar pigs than those in barren pens (Table 2). However, pigs in enriched pens only spaced further away from each other than pigs in barren pens during the batches with a maximum ambient temperature around 21.5°C (Figure 4: Jan, Mar, Oct), whereas in the batch with a maximum ambient temperatures of 29.7°C (Jun) there was no significant difference between housing conditions (Figure 4). This interaction between housing condition and batch, which is confounded with temperature, was apparent for the distance to the familiar pig (*P* = 0.08) and to unfamiliar pigs (*P*<0.01). The relative distance to unfamiliar pigs was smaller, which means that pigs in enriched pens were relatively closer to unfamiliar pigs than to the familiar pig as compared to pigs in barren pens (Table 2). Pigs in barren pens lay twice as much in body contact with familiar or unfamiliar pigs than pigs in enriched pens (Table 2).

**Table 2:** Effect of barren and enriched housing on the distances between pigs.

	Barren	Enriched	*P*-value
Distance to familiar pig	115±3	136±3	<0.001
Distance to unfamiliar pigs	151±3	165±3	<0.001
Relative distance	1.4±0.04	1.3±0.04	0.04
Body contact with familiar pig	18±1.3	11±1.3	<0.001
Body contact with unfamiliar pigs	28±1.4	14±1.4	<0.001

Absolute distances are in cm, whereas the relative distance equals the number of times that a pig is further away from the unfamiliar pig compared to the familiar pig. Body contact is expressed in percentage of observations. Values are LSmeans ± SEM.

### Indirect genetic effects

**Figure 5:**
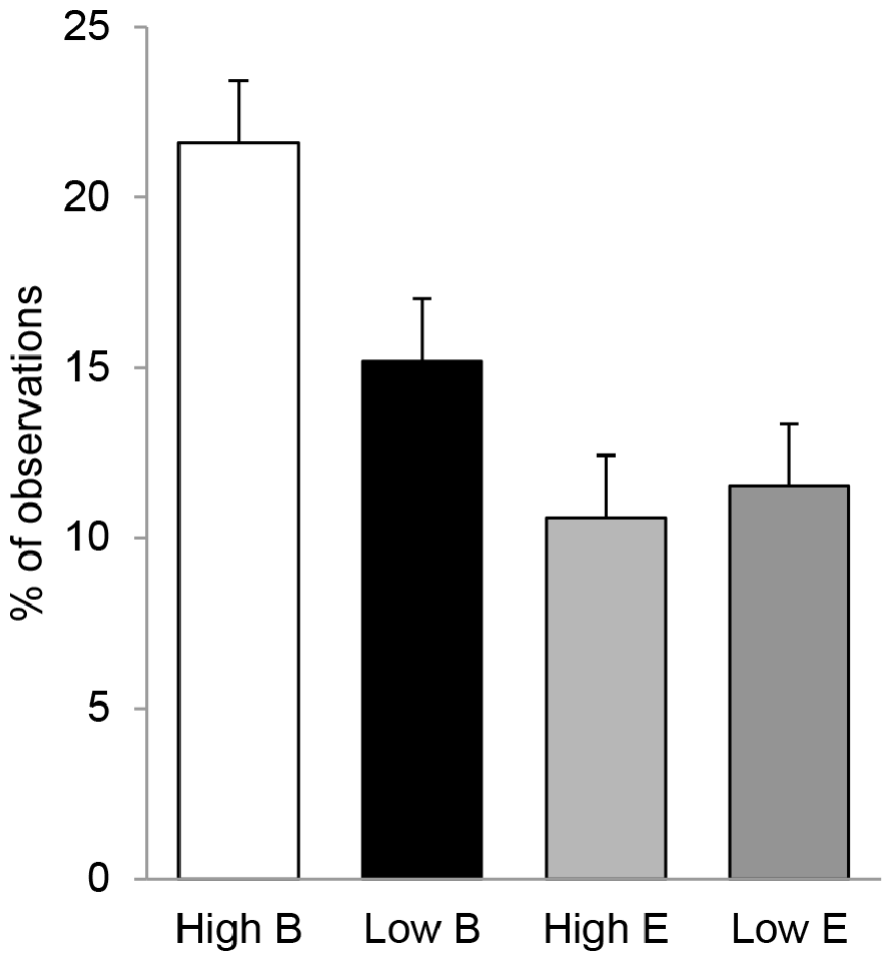
Genotype-environment interaction. Percentage of observations that a pig was lying in body contact with the familiar pig, given for pigs which were genetically selected for either high or low IGEg, and housed in barren (B) and enriched (E) pens.

There were no significant effects of selection on ‘indirect genetic effect for growth’ (IGEg) on any of the measures on spatial integration (all *P* >0.10). The genotype by environment set-up, however, tended to interact for lying in body contact with the familiar pig (*F*_1,126_ = 2.99; *P* = 0.09). Pigs which were genetically selected to have a positive effect on the growth rate of their group members (high IGEg pigs) were lying most frequently in body contact with their familiar pig when they were housed in barren pens. In contrast, of the four treatment groups (IGEg × housing condition), high IGEg pigs were lying least frequently in body contact with the familiar pig when they were housed in enriched pens (Figure 5).

## Discussion

We aimed to assess the relationship between behaviour, skin lesions, and plasma haptoglobin of pigs and their spatial integration during social conflict. Pigs clearly distinguished between familiar and unfamiliar pigs over the 24 h period after mixing, whereby they remained closer to the familiar pig. The results highlight that reduced aggression does not necessarily equate to reduced social tension, which may be reflected by large spatial distances. It is therefore important to consider a spectrum of parameters before conclusions are drawn upon the contribution of an animal to the functioning of a group.

### Characteristics of pigs with a large inter-individual distance

Factor 1 in the principal component analysis described sociality, whereby pigs with high factor 1 scores were inactive pigs with a high number of skin lesions, high body weight, and little social nosing behaviour. These pigs tended to space further away from all other pigs irrespective of familiarity. It seems plausible that these pigs were exhausted of fighting and were therefore less active. As a result of fighting and higher body weight, they may have had an increased body temperature [40], which might make them space further away from others. This withdrawn position may have led to less social interactions in the form of social nosing. These pigs might also have been unsuccessful in their fights and consequently might not have been allowed in close proximity [41]. If aggressiveness is the underlying cause of large inter-individual distances than selection against aggressiveness [42] might change inter-individual distances. It could also be that these pigs were dominant. Dominant pigs may keep a larger portable (personal) space than subordinates [43] which may reflect their own choices or the unwillingness of other animals to closely approach them [13]. This high dominance status would be in line with the high body weight [44], and high amounts of aggression as reflected in the skin lesions [18]. If dominance is underlying the differences in distances, than a more rapid establishment of dominance relationships might lead sooner to social cohesion. Irrespective of the underlying cause, the response of the ‘antisocial’ pigs seemed to hamper the social cohesion of the group of pigs. For future studies it would be worthwhile to include tests on aggressiveness and dominance status.

### The role of aggression

Factor 2 described involvement in aggressive behaviour given and received. Factor 2 did not have any significant effect on the measures of spatial integration, which indicates that studying aggression alone may miss important aspects of social conflict. A potential reason why factor 2 did not relate to any of the measures of spatial integration might be the simultaneous inclusion of given and received aggression in the PCA. The amounts of aggressive behaviours that are given and received are correlated [18]. Giving aggression may provoke a different behavioural repertoire than receiving aggression, and this may be reflected in the inter-individual distances measured at other time points than the aggressive act. That these different types of involvement in aggression loaded equally on factor 2 may have caused that factor 2 did not reveal any significant effects on the spatial integration. More detailed observation of aggressive behaviour, including the time frame directly after regrouping, might give more clarity on the relationship between aggression and spatial distances.

### The role of social nosing

Factor 1 comprised social nosing behaviour, in counter direction to measures on inactivity, aggression, and body weight. Social nosing in pigs may amongst others contribute to recognition and affiliation, and may result in acceptance of a conspecific within the group [41], [45]. From the current observations, it was not possible to determine whether pigs were nosing a conspecific to recognize the individual, or to express affiliative behaviour. Both may be true, as pigs can easily recognize and remember familiar conspecifics [41], yet 20% of the social nosing was directed towards the familiar group mate. Studies from other species indicate that both recognition and affiliative behaviour may have a positive effect on social cohesion [5],[46]. Most studies on affiliative behaviour, however, are based on stable social groups, whereby affiliative behaviour may for example function as a tool to reconsolidate [8]. The current study concerned newly formed groups, and the social nosing towards unfamiliar conspecifics might therefore be more likely related to recognition and getting acquainted to each other, which may include sorting out dominance relationships [5], [46]. The establishment of dominance relationships may depend on amongst others aggressiveness and physiological differences such as body weight [44], [49], [50] but see [51]. Pigs with a low factor 1 score were lighter, had less skin lesions, and were more active. These small pigs might have actively avoided fights, or may have gained their rank without the necessity to fight as a result of their low weight compared to others [49]. The pigs with a low factor 1 score gave more social nosing and were more likely to approach other pigs, including unfamiliar ones. This suggests that these pigs may cope differently with social conflict than pigs with other predominant factor scores.

### The role of haptoglobin

Factor 3 described the increase in plasma haptoglobin concentrations. Haptoglobin concentrations may be heightened with infection or inflammation (e.g. [31], [32], in pigs [34], [52]), but has also been increasingly explored as a biomarker of stress [30], [31], [35], [53]. The suggested pathway is that, in response to stress, the sympatho-andrenal axis and the hypothalamic-pituitary-adrenal axis would release catecholamines and glucocorticoids, respectively, and that these activate acute phase proteins such as haptoglobin in the liver [30]. Haptoglobin has been found to increase amongst others during road transport [33], [35], [53], [54], and with regrouping pigs [55], although a number of studies found no effect of these events on haptoglobin concentrations (in pigs [56], [57], [58]). Regrouping may coincide with an increased immune response [59], which is mainly due to skin lesions. Skin lesions were, however, in the current study unrelated to the increase in haptoglobin. This suggests that the significant increase in haptoglobin after regrouping as compared to baseline values was due to an overall stress reaction of the pigs which could not be attributed to skin lesions. Factor 3 had no influence on the spatial integration between pigs, but did interact with factor 2. The largest relative distances, thus stronger distinction between familiar and unfamiliar pigs, was seen in pigs which were much involved in aggressive behaviour and had a strong increase in haptoglobin. As a response to the either physical or physiological stressful situation, these animals may have remained closer to the familiar conspecific, as was reflected in the high frequency of lying in body contact with the familiar pig. The presence of a familiar conspecific may have a stress-buffering effect due to social support, also in pigs [60], [61], [62]. Pigs have been reported to prefer lying beside a familiar pig for up to six days after mixing, especially in more aggressive groups [63], [64]. The higher Δhaptoglobin levels, as well as the tendency to remain in close proximity to the familiar conspecific, suggests that these animals experienced more stress, either by unsuccessful fights or by a heightened vulnerability to stress [22].

### Enriched housing conditions

Apart from the individual pig characteristics that may influence the distance between pigs, the enriched housing caused a considerable increase in the distances between animals, regardless of familiarity. The relative distance towards unfamiliar pigs was smaller in enriched pens, which may have been due to the limited pen dimensions imposing a maximum distance that could be reached, i.e. the pig pair may had reached the maximum distance that they could space away from the four unfamiliar pigs. Activity in general will increase body temperature, and the insulating properties of straw may preserve body heat more than concrete [65], [66], which might cause animals to space further apart. The increased ambient temperature in the month of June, however, hardly affected the distances (Figure 4), which suggests a role for other factors. The increased absolute distance might, however, also indicate increased social tension or aggression in enriched pens. Straw-enrichment may increase aggressive behaviour [67] due to the availability of defensible resources such as fresh straw or a dry lying area [27], [68]. A reduction, or lack of impact on aggression from straw enrichment has however also been reported [67], [69]. In an earlier study we reported that the pigs on straw did not show more aggressive behaviour, but did have more skin lesions as a result of enriched housing [27], which might be related to increased activity [27], [69]. Environmental enrichment may direct the attention towards the physical environment rather than to the social environment [70], also when straw is not novel to pigs, and this might have caused pigs to spend less time in social interaction and thus potentially resulted in larger distances between pigs.

### Indirect genetic effects

Pigs that were selected on high IGEg, which is a positive genetic effect on the growth of pen mates, spent almost twice as many observations in body contact with the familiar pig than pigs from other treatment groups. The inter-individual distance between animals may decrease with genetic selection for social traits, as has been observed in Japanese quail (*Coturnix japonica*) selected for social reinstatement behaviour [71]. The distance may decrease further in novel and potentially stressful environments [71]. Stress is likely to be greater in barren housed animals as compared to enriched housed animals [72], [73]. As argued above, being in close contact with a familiar conspecific may reduce stress. Previous studies suggested that high IGEg pigs cope better with stressful situations [27], [61], [74], [75]. It might be that this capacity to cope with stress relates to a higher tendency to give or seek social support.

### An integral approach to social conflict

The combination of behavioural and physiological data suggests that spatial integration depends mostly on aggression, sociability, and stress vulnerability. The distances and amount of body contact between unfamiliar pigs could only be observed on pair level, which was a restriction to the study design. The familiar pig pairs, however, commonly stayed in close proximity to each other, and thus their average distance to the other pigs is a reasonable approximation of their individual distance to unfamiliar pigs. Aggression as a single parameter failed to indicate spatial integration between pigs, but in combination with haptoglobin it revealed pigs that spent more time in body contact with the familiar pig and spaced relatively further away from unfamiliar pigs. The social cohesion was hampered by animals with a high factor 1 score, indicating much skin lesions, high body weight, little social nosing, and inactivity. It is unclear whether these pigs spaced away from others due to dominance status, exhaustion, or high body temperature. Contrary, pigs that showed much social nosing were low in body weight, had few skin lesions, were more active, and were more prone to approach familiar and unfamiliar pigs. Genetics and housing conditions may affect inter-individual distances between pigs, whereby straw housing may lead to a larger portable space. If we would like to improve spatial integration between unfamiliar animals upon encounter, this would not necessarily require animals with little aggression per se, but would rather require animals that cope well in a group, for example by rapid establishment of dominance relationships. Absence of fighting is not the same as being free of social tension, and therefore an integral approach may be necessary when assessing animal welfare.

## Supporting Information

Table S1 **Data file by observation (n = 5317).**
(XLSX)

Table S2 **Data file by pig (n = 374).**
(XLSX)
